# Nursing home staff’s perspective on end-of-life care of German nursing home residents: a cross-sectional survey

**DOI:** 10.1186/s12904-019-0512-8

**Published:** 2020-01-03

**Authors:** Anke Strautmann, Katharina Allers, Alexander Maximilian Fassmer, Falk Hoffmann

**Affiliations:** 1 Department of Health Services Research, Carl von Ossietzky University of Oldenburg, Oldenburg, Germany; 2 Department of Health Services Research, School VI - Medicine and Health Sciences, Carl von Ossietzky University of Oldenburg, Ammerländer Heerstr. 114–118, D-26129 Oldenburg, Germany

**Keywords:** Nursing home residents, End-of-life care, Nursing staff, Survey

## Abstract

**Background:**

Nursing homes are becoming more important for end-of-life care. Within the industrialised world, Germany is among the countries with the most end-of-life hospitalizations in nursing home residents. To improve end-of-life care, investigation in the status quo is required. The objective was to gain a better understanding of the perspectives of nursing home staff on the current situation of end-of-life care in Germany.

**Methods:**

A cross-sectional study was conducted as a postal survey among a random sample of 1069 German nursing homes in 2019. The survey was primarily addressed to nursing staff management. Data was analyzed using descriptive statistics. Staff was asked to rate different items regarding common practices and potential deficits of end-of-life care on a 5-point-Likert-scale. Estimations of the proportions of in-hospital deaths, residents with advance directives (AD), cases in which documented ADs were ignored, and most important measures for improvement of end-of-life care were requested.

**Results:**

486 (45.5%) questionnaires were returned, mostly by nursing staff managers (64.7%) and nursing home directors (29.9%). 64.4% of the respondents rated end-of-life care rather good, the remainder rated it as rather bad. The prevalence of in-hospital death was estimated by the respondents at 31.5% (SD: 19.9). Approximately a third suggested that residents receive hospital treatments too frequently. Respondents estimated that 45.9% (SD: 21.6) of the residents held ADs and that 28.4% (SD: 26.8) of available ADs are not being considered. Increased staffing, better qualification, closer involvement of general practitioners and better availability of palliative care concepts were the most important measures for improvement.

**Conclusions:**

Together with higher staffing, better availability and integration of palliative care concepts may well improve end-of-life care. Prerequisite for stronger ties between nursing home and palliative care is high-quality education of those involved in end-of-life care.

## Background

Demographic change is challenging society across the world. In western industrialised countries especially birth rates are decreasing while the steady improvement of an already highly developed level of medicine allows the older people to grow ever older [1]. It generally is estimated that approximately 12–17% of the aging adults living in high-income countries are in need of regular care [2].

In Germany around 3.5 million people were care-dependent in 2017 [3] and, therefore, received benefits either from the social or the private long-term care insurance. Among these, nearly 750,000 were older than 65 years and lived in nursing homes [4]. More than half of the nursing homes in Germany are run by non-profit organizations, approximately 40% by private for-profit organizations, and the remainder are in public ownership, usually run by municipalities [5]. With the demographic change continuing, the number of older care-dependent people is estimated to increase up to 4.5 million by 2060 [6]. Hence, the demand for nursing care is expected to rise, too. At the same time, the demographic change is also likely to negatively affect the availability of skilled nursing staff on the job market [7].

Many older people are moving into nursing homes at the end of their lives, often due to dementia or the aftermath of a stroke [8]. In many cases, they are in need of high-quality end-of-life care. High-quality end-of-life care is often characterized by special attention being paid to individual needs [9] and symptoms and pain being dealt with adequately [10–14]. The fear of undertreatment, especially regarding pain and other distressing symptoms, seems to be present among those faced with the end of their lives, their relatives, but also the nursing staff and physicians [15, 16]. However, not only undertreatment, but also overtreatment can become a distressing issue at the end of a person’s life [17, 18]. Overtreatment can reveal itself in many different forms, with unnecessary end-of-life hospitalization being one of them [19].

These hospitalizations might lead to adverse effects, which can include temporary and permanent cognitive or functional decline [20–24] as well as becoming part of the cascade iatrogenesis [25–27]. Furthermore, every hospital transfer interrupts care consistency, which may well place individuals at the risk of inappropriate medication [28, 29]. It has been shown that individuals with already existing cognitive and functional deficits as well as older age are at greater risk of suffering from such effects [25, 30, 31]. Nursing home residents, who usually fulfil at least two of these criteria, are therefore a high-risk group for negative health-related consequences arising from end-of-life hospitalization.

A systematic review revealed that about every third nursing home resident worldwide experiences hospitalization during their last month of life [32]. In Germany, more than half of the institutionalized population is hospitalized in the last month of life [32] and is, therefore, at risk of having to suffer from adverse health effects as resulting from these hospitalizations. About one third of all German nursing home residents die in a hospital [33, 34]. However, reasons for this comparably high proportion of burdensome end-of-life hospitalizations in Germany have not yet been investigated.

Therefore, the aim of this study was to investigate the perspective of nursing home staff on end-of-life care, particularly their opinion on end-of-life hospital transfers, advance directives (ADs), and most important problems of end-of-life care as well as measures for improvement.

## Methods

### Study design and data collection

In this cross-sectional study data was collected with the help of a postal survey. For this survey 1121 nursing homes were randomly selected from the approximately 11,200 nursing homes in Germany. Nursing homes caring mostly for children and young adults, mentally and physically disabled residents, ventilated residents, and residents in a persistent vegetative state were excluded (*n* = 52). The survey was sent to the remaining 1069 nursing homes, first in January 2019. Each of these nursing home received one questionnaire that was primarily addressed to the nursing staff management. A reminder was sent approximately a month later, in February 2019.

Several strategies, such as using a short questionnaire, follow-up contact, providing a second copy of the questionnaire at follow-up, personalized letters, and academic origin of the study were applied. These strategies have a positive effect on response as shown by a Cochrane review [35]. All data was collected anonymously.

The data of all questionnaires was entered into a database by one researcher and independently validated by a second researcher. Differences between both researchers were resolved either by discussion or by involving a third researcher.

### The questionnaire

The four-page questionnaire was developed by a multidisciplinary research team (see Additional file 1). It was based on a study investigating the medical care of nursing home residents as addressed to general practitioners (GPs) within the scope of the HOMERN (Hospitalization and emergency department (ED) visits of nursing home residents) project [36]. Several questions from the original questionnaire, which was designed for GPs, were substituted with questions designed for nursing home management. Most questions and the methods used for this as well as for the study addressed to GPs [36] were similar. Hence, results of both studies will be compared in the discussion.

The questionnaire was subdivided into three different parts. Each part dealt with one of the following issues: a) medical care provision in hospitals and EDs, b) care provision at the end of life, and c) care delivered by physicians and allied health professionals. The present study mainly focuses on aspects dealing with end-of-life care.

Firstly, participants were asked to estimate the proportion of nursing home residents with documented AD. Furthermore, they were requested to judge how many of the ADs of the German nursing home residents contain information about hospital transfers and in how many cases the resident’s wishes documented in the AD were not taken into account. Secondly, respondents were asked to rate five different statements regarding common practices and potential deficits in end-of-life care on a 5-point Likert scale (0 = totally disagree to 4 = totally agree). This also included one question about specialized outpatient palliative care (SAPV) in Germany. SAPV is a nationwide programme available for patients with limited life expectancy being carried out by multidisciplinary and multi-professional teams specialized in palliative care [37].

Thirdly, respondents were requested for estimations of the proportions of nursing home residents dying in hospital and whether these proportions have decreased, increased or have remained unchanged over the past decade. Lastly, participants were asked to indicate their perception of the end-of-life care nursing home residents receive, either as rather poor or rather good. Those who indicated a rather poor quality of end-of-life care were invited to describe what they perceive as the most important measure for improving end-of-life care (free text).

Additionally, sociodemographic data of the respondents, such as age, sex, current position, and the amount of time spent in that position were requested. Respondents were also asked to provide the following information on the associated nursing homes: their sponsorship; number of beds; and location, including details about the federal state, the number of inhabitants (rural ≤20,000; semi-urban > 20,000- ≤100,000; urban > 100,000 inhabitants), and the distance to the next hospital with an ED.

### Statistical analysis

Descriptive statistics were computed. For categorial data frequencies were calculated. Continuous data are presented as mean with standard deviation (SD), median and interquartile range (IQR).

Since not all respondents answered every question in the questionnaire, the analyses were restricted to subjects with no missing values given in the respective questions.

All statistical analyses were carried out using SPSS Version 25.

Free-text answers were summarized and allocated to previously determined categories. Categories were similar to those used in the aforementioned study involving GPs [36], but slightly adjusted to the answers given in this survey. Allocation was conducted by one researcher and independently checked by a second. Discrepancies were either solved by discussion or by involving a third researcher.

The study received a waiver from the local medical ethics committee of the Carl von Ossietzky University of Oldenburg (no. 2018–147).

## Results

### Characteristics of the respondents and the nursing homes

In total 486 questionnaires were included in the analysis (response: 45.5%). Respondents had a mean age of 48.0 years (SD: 9.8) and were mostly female (71.0%). Almost two thirds (64.7%) were nursing staff managers and 29.9% were nursing home directors. On average, respondents had around 9.7 years (SD: 8.0) of work experience in their current position (Table 1).

**Table 1:** Characteristics of the respondents

Characteristics	in % (n)
Respondents	
Age in years (*n* = 465)	
≤35	14.4 (67)
36–45	23.2 (108)
46–55	34.8 (162)
> 55	27.5 (128)
Mean (± SD)	48.0 (± 9.8)
Sex (*n* = 476)	
Male	29.0 (138)
Female	71.0 (338)
Position (*n* = 479)	
Nursing staff manager	64.7 (310)
Nursing home director	29.9 (143)
other	5.4 (26)
Work experience in current position in years (*n* = 474)	
≤5	39.0 (185)
6–10	25.1 (119)
11–15	15.2 (72)
16–20	10.8 (51)
21–25	5.1 (24)
> 25	4.9 (23)
Mean (± SD)	9.7 (± 8.0)
Nursing homes	
Size – number of beds (*n* = 478)	
≤50	18.6 (89)
51–100	51.0 (244)
101–150	23.4 (112)
151–200	5.0 (24)
> 200	1.9 (9)
Mean (± SD)	89.1 (± 47.5)
Sponsorship (*n* = 469)	
Non-profit	52.7 (247)
Private	39.2 (184)
Local	8.1 (38)
Area (*n* = 465)	
North Germany^a^	22.4 (104)
West Germany^b^	35.9 (167)
East Germany^c^	15.1 (70)
South Germany^d^	26.7 (124)
Location (*n* = 461)	
Rural (≤20,000 inhabitants)	51.6 (238)
Semiurban (> 20,000 - ≤100,000 inhabitants)	28.4 (131)
Urban (> 100,000 inhabitants)	20.0 (92)
Distance to the next hospital with an emergency department in km (*n* = 419)	
< 1	4.8 (20)
1–5	45.6 (191)
6–10	21.2 (89)
11–15	14.3 (60)
16–20	7.6 (32)
21–25	3.1 (13)
> 25	3.3 (14)
Mean (± SD)	8.5 (± 7.8)

*SD* standard deviation

^a^ federal states Bremen, Hamburg, Mecklenburg-Western Pomerania, Lower Saxony, Schleswig-Holstein

^b^ federal states Hesse, North Rhine-Westphalia, Rhineland-Palatinate, Saarland

^c^ federal states Berlin, Brandenburg, Saxony, Saxony-Anhalt, Thuringia

^d^ federal states Baden-Wuerttemberg, Bavaria

The participating nursing homes had on average 89.1 beds (SD: 47.5). The number of beds ranged from 4 to 403 per home. More than half (52.7%) of the nursing homes had a non-profit, 39.2% a private and 8.1% a local sponsorship. More than half (51.6%) of the responding nursing homes were located in rural areas, 28.4% in a semiurban and 20.0% in an urban area. On average, the distance to the next hospital with an ED was 8.5 km (SD: 7.8). More than half (50.4%) of the participating nursing homes were separated from an ED by 5 km or less.

### Advance directives and hospitalization at the end of life

The respondents stated that, in their estimation, approximately 45.9% (SD: 21.6) of the residents held an AD. They also estimated that in 29.4% (SD: 27.2) of the cases these AD contain information regarding hospital transfers at the end of life. According to the respondents’ perception, in more than a quarter (28.4%; SD: 26.8) of all residents with an AD the wishes documented in these directives are not taken into account (Table 2).

**Table 2:** Assessment of the current situation in German nursing homes

	Mean in % ± SD(Median; IQR)
Estimated proportion of residents with an advance directive (*n* = 476)	45.9 ± 21.6 (40.0; 30.0–60.0)
Estimated proportion of advance directives that are informative regarding hospital transports at the end of life (*n* = 457)	29.4 ± 27.2 (20.0; 10.0–50.0)
Estimated proportion of advance directives that are not taken into account in accordance with residents’ wishes (*n* = 423)	28.4 ± 26.8 (20.0; 5.0–50.0)
Estimated proportion of residents dying in hospital (*n* = 462)	31.5 ± 19.9 (30.0; 15.0–50.0)
Estimation whether the proportion of residents died in hospital decreased, remained unchanged or increased over the last 10 years (*n* = 464)	in % (n)
Decreased	55.4 (257)
Remained unchanged	24.8 (115)
Increased	19.8 (92)
Overall rating of end-of-life care in nursing homes (*n* = 469)	
Rather poor	35.4 (166)
Rather good	64.6 (303)

Respondents estimated that 31.5% (SD: 19.9) of the nursing home residents die in hospital. More than half (55.4%) of the respondents think that the proportion of nursing home residents dying in hospital has decreased over the past 10 years.

### End-of-life care

**Fig. 1:**
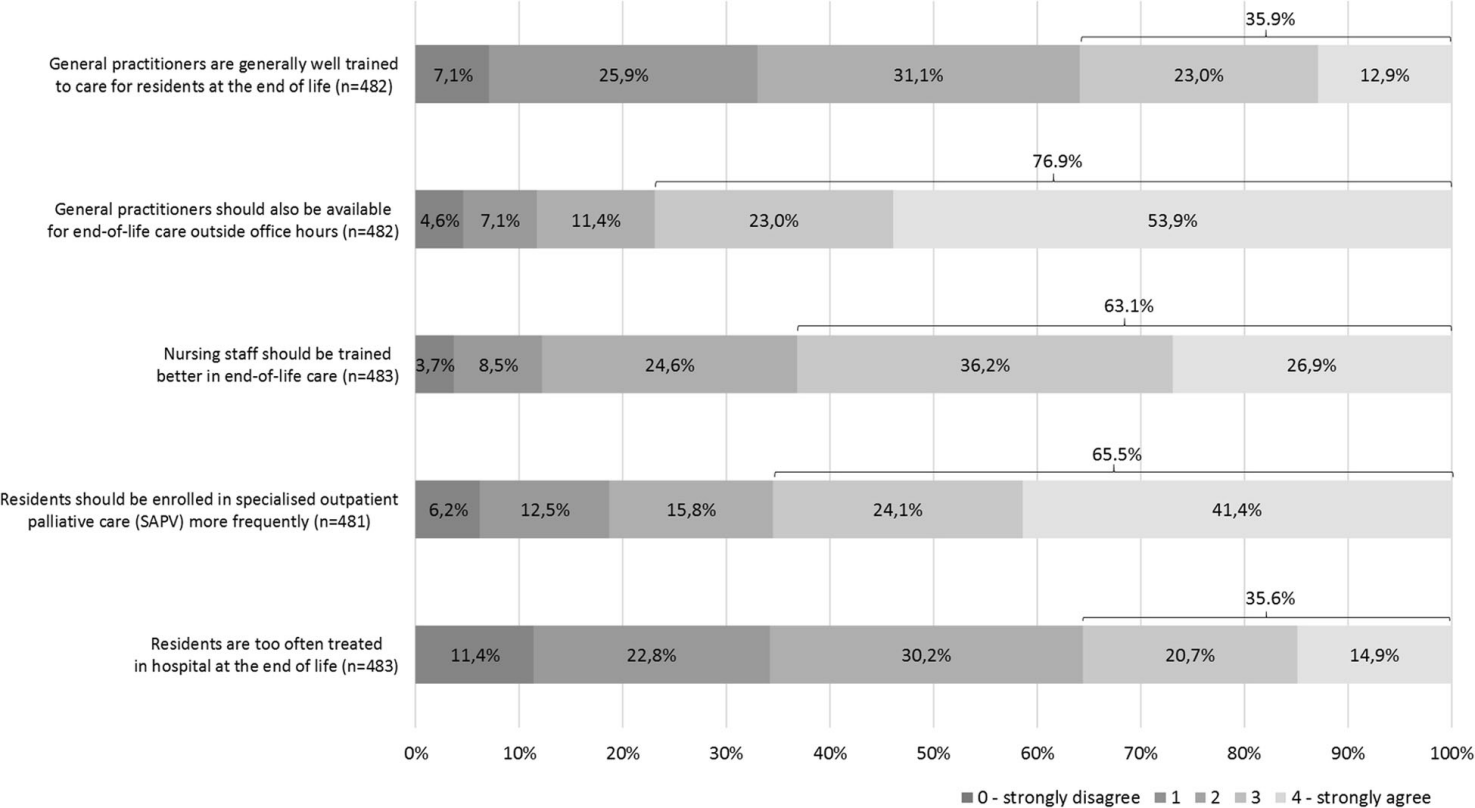
Perspectives of the respondents on status quo and potential deficits of end-of-life care

About a third (35.6%) of the respondents agreed that nursing home residents are treated in hospitals too often at the end of life, while approximately another third (34.2%) of the respondents was convinced of the opposite. Nearly two thirds (65.5%) answered that residents should be enrolled in SAPV more frequently. Around two thirds (63.1%) of the respondents see a need for further education of nurses working in end-of-life care, while 35.9% think that GPs are already well-trained regarding end-of-life care. More than three quarters (76.9%) of the respondents agreed that GPs should be available after office hours in the case of end-of-life care-issues (Fig. 1).

Overall, approximately one third (35.4%) rated end-of-life care rather poor.

### Measures for improvement

Overall, 183 participants elaborated on what they see as the most important measure for improving end-of-life care mentioning a total of 334 items. For 320 of these items a total of 15 categories were built, the remaining 14 items were so diverse that they could not be allocated to one of the 15 categories. The four most frequently mentioned categories were “more nursing staff” (28.4%), “better integration and better availability of palliative and hospice care in nursing homes” (17.4%), as well as “better qualification of the nursing staff” (12.9%), and “closer involvement of the GPs” (9.3%). The remaining categories were mentioned in 0.9–5.1% of the cases and included, for example, measures like better communication and cooperation, better availability of medication, strengthening the residents’ wills/ advance care planning (ACP), less hospital treatment, and better and clearer legislation regarding both, conducting and forgoing medical treatment at the end of life (Table 3).

**Table 3:** Most important measures to improve end-of-life care (*n* = 334)

More nursing staff (incl. Less staff turnover, more time per resident)	28.4%
Better integration and better availability of palliative and hospice care in nursing homes	17.4%
Better qualification of the nursing staff (incl. Training in palliative care)	12.9%
Closer involvement of the general practitioners	9.3%
Better qualification of the general practitioners (incl. Training in palliative care)	5.1%
Better communication and cooperation	3.6%
Better availability of medication (especially regarding pain management)	3.6%
Closer involvement of relatives	3.0%
Strengthening the residents’ will/ advance directives/ Advance care planning	2.7%
Closer involvement of other staff (volunteers, psychosocial counselling)	2.7%
Less hospital treatment	2.1%
Legal certainty of nurses’ and physicians’ procedures	1.5%
Expansion of the responsibilities of the nursing staff	1.5%
Increased payment	1.2%
Reduction of administrative tasks and documentation, better organizational structures	0.9%
Other items	4.2%

## Discussion

### Findings and comparison with the literature

In general, end-of-life medical care of nursing home residents was perceived as rather poor by more than one third of the respondents (35.4%). The prevalence of ADs was estimated at 45.9%. Nearly two thirds (63.1%) of the respondents think that nursing home staff should be educated better regarding end-of-life care and about a third (33.0%) suggest more training for end-of-life care for GPs. The proportion of residents dying in hospital was estimated at 31.5%. More than half (55.4%) assume a decrease in in-hospital deaths over the past 10 years.

In fact, a German study published in 2006 found that 28.9% of the 792 nursing home residents included in the study died in hospital [33], while another German study, published in 2019, found a proportion of 29.5% in-hospital deaths among around 67,000 German nursing home residents [34]. These figures suggest that the proportions of in-hospital deaths have remained more or less constantly high over the past decades, while the international literature shows inconsistent findings [32]. End-of-life hospitalization, though, is often linked to adverse health effects [20–29]. Hence, many older people prefer dying at home or in the nursing home [38].

A possible way of fostering a decrease in end-of-life hospitalization could be the further promotion and development of ACP. A systematic review has already shown that hospitalization can be decreased through ACP [39]. ADs are usually part of ACP. Respondents in this study and GPs from the above mentioned study [36] estimated that around 45.9 and 36.8% of the residents, respectively, hold an AD. These estimations are similar to previous studies which have shown that around 12–37% of the German nursing home population hold an AD [38, 40, 41]. Respondents also estimated that only 29.4% of the ADs contained information on end-of-life hospital transfers. Literature from the United States and Canada suggests that around 4–23% of the nursing home residents have do-not-hospitalize orders [42–45]. However, the American and the Canadian health care systems can hardly be compared to the German one. When asked for the most important measure to improve end-of-life care, only around 5.0% of the respondents of this study and 2.1% of the GPs [36] mentioned strengthening the resident’s will or supporting the use of ADs. This could be due to the fact that ADs are often perceived as recommendations rather than binding instructions [46]. Moreover, a study from Germany has shown that most ADs either contained only very little information or were invalid [40]. Results from a Dutch study show, though, that even with only around 5% of nursing home residents holding ADs, end-of-life hospitalization was only around just 8% during the last month of life [47]. A possible reason may be that all care homes included in the aforementioned study have palliative care goals [47]. The sole use of ACP and ADs may have positive effects on the quality of end-of-life care; however, when embedded into palliative care concepts the positive effects of measures such as ACP and ADs could be manifold.

End-of-life care was rated as rather poor by 35.4% of the respondents of this study as well as by 53.8% of the GPs of the recently conducted and comparable study [36] suggesting that there still is room for improvement. On the one hand the respondents of this study see potential for improvement in increasing the number of residents being enrolled in SAPV. These programmes have been available in Germany since 2007 and a study has already proven their effectiveness in reducing end-of-life hospital transfers and emergency physician consultations [48]. However, their utilization still seems to lack practicability, especially in rural areas [48]. Moreover, palliative care in Germany is so far mostly addressed to people with malignant diseases. The proportion of cancer patients among those receiving palliative care is more than 90% [49], but in nursing home residents dementia, cardiovascular diseases, fractures, and infections are far more common [50, 51]. This further supports the respondents’ perception of residents being enrolled in palliative care programmes too infrequently.

In our recently conducted study on GPs, especially GPs with palliative training rated end-of-life care as rather poor [36]. This, on the other hand, suggests that strengthening the ties between palliative care and nursing home care is an important measure to improve end-of-life care. Studies from the US have already shown that palliative care consultations can contribute to less end-of-life hospitalization [52, 53]. Furthermore, countries fostering palliative care concepts in nursing homes have considerably lower prevalences of end-of-life hospitalization [47, 54–56]. However, even within Europe, there is a broad range of health care systems and the integration of palliative care in nursing homes is very diverse [57]. Thus, ‘good practice’ concepts of palliative care integration in nursing homes from other countries cannot be applied without changes. Every country has to develop solutions to fit their own, specific health care system framework.

The key to further implementation and use of palliative care concepts seems to be educational training as literature has shown that nurses in German nursing homes considerably lack education in palliative care [58]. Respondents of this study as well as GPs [36] have also suggested that a better qualification and education of the nursing staff could significantly improve end-of-life care. A better understanding of the different processes that occur at the end of life and around dying could lead to a more adequate response to residents’ needs and concerns at this particularly vulnerable stage of life. Additionally, further qualification could lead to an expansion of responsibilities for the nursing staff. With better education and broader responsibilities, pain and symptom management, for example, could be improved. Studies have already investigated ways to better educate those involved in end-of-life care, but a systematic review has shown that these studies and interventions, unfortunately, lack quality [59]. Hence, further research needs to be conducted in order to develop and evaluate high-quality training for end-of-life care.

### Strengths and limitations

The aim of this study was to gain an overview of perceptions of German nursing home staff on end-of-life care. Therefore, some of the data have to be interpreted cautiously, especially the estimations the participants were requested for as it is not clear how valid these estimations are. As the majority of respondents were either nursing staff managers or nursing home directors, their answers may have been biased in order to represent their own nursing home more positively. However, it was emphasized that all data was collected anonymously and the proportion of estimated in-hospital deaths fits very well into the literature. Furthermore, for most of the information we were interested in, to date hardly any robust data and literature from Germany can be found. In order to get more detailed and robust data supporting the overview gained here, further prospective studies should be conducted.

The argumentation of this study implies that end-of-life hospitalization is a negative event. There may be situations in which nursing home care is too limited to adequately deal with residents’ health issues and where hospital treatment is needed. However, literature suggests that end-of-life hospitalization often results in adverse effects [20–29] and that the majority of nursing home residents prefers to die in the nursing home rather than in a hospital [38]. Moreover, as many as two out of three of all hospitalizations in nursing home residents are considered potentially avoidable [19, 60–63]. Therefore, not every hospital transfer, but certainly inappropriate and unnecessary ones can be considered negative events. The study itself did not distinguish appropriate from inappropriate or avoidable hospitalization.

A major strength of this study is the large sample of nearly 500 participants and a high response (45.5%).

Questionnaires were, in most cases, not answered by those directly involved in end-of-life care of the nursing home residents, but by nursing staff managers or nursing home directors. However, nursing staff managers and nursing home directors are more likely to have a broader and more comprehensive overview of the common practice and of the potential deficits of the end-of-life care in their nursing homes.

## Conclusion

Overall, more than one third of the participants of this study consider end-of-life care of German nursing home residents rather poor. Germany is among the countries with the highest amount of end-of-life hospitalization in the western industrialised world. Hence, improvement needs to be made and one way may be to properly document detailed information on this topic in the form of ADs. Another way of improving end-of-life care may be supporting the utilization of palliative care as well as spreading palliative care concepts. This may be reached through further training of nurses and physicians, but more research is needed to develop highly efficient and well evaluated trainings in the context of the German health care system. With better training and, in turn, expansion of the nurses’ responsibilities as well as wider availability of palliative care and higher staffing, end-of-life medical care of German nursing homes may well improve.

## Supplementary information

**Additional file 1.** This file contains the original version of the questionnaire used for this study.

## Data Availability

The datasets generated during and/or analysed during the current study are available from the corresponding author on reasonable request.

## Abbreviations

ACP: Advance Care Planning
AD: Advance Directives
ED: Emergency Department
GP: General Practitioner
HOMERN: “Hospitalization and emergency department visits of nursing home residents”-Project
IQR: Interquartile Range
SAPV: Specialized Outpatient Palliative Care Programme
SD: Standard deviation
