# Supplementary material for: Nursing home staff’s perspective on end-of-life care of German nursing home residents: a cross-sectional survey
Source: BMC Palliat Care. 2020 Jan 3;19:2. doi: 10.1186/s12904-019-0512-8 (PMC6942381; doi:10.1186/s12904-019-0512-8)
Supplement: Supplementary file 1 — Additional file 1. This file contains the original version of the questionnaire used for this study. [file 12904_2019_512_MOESM1_ESM.pdf]

Einschätzung zur medizinischen Versorgung von Pflegeheimbewohnern

HOMERN

Bei Rückfragen wenden Sie sich bitte an:

Carl von Ossietzky universität Oldenburg | Universität Bremen

Alexander Fassmer | Carl von Ossietzky Universität Oldenburg, Department für Versorgungsforschung, Ammerländer Heerstr. 114 - 118, 26129 Oldenburg
Telefonnr.: 0441 798-2766 | E-Mail: HOMERN@uni-oldenburg.de

Im folgenden Fragebogen geht es um die Beurteilung der medizinischen Versorgung von Bewohnern aus Sicht der Pflegeheime. Der hier verwendete Begriff „Krankenhaustransport" umfasst sowohl stationäre Aufenthalte als auch ambulante Notaufnahmebesuche.

***Zu Gunsten der besseren Lesbarkeit wird im Fragebogen ausschließlich die männliche Form verwendet (z.B. Heimbewohner, Hausärzte). Frauen sind dabei selbstverständlich in gleicher Weise angesprochen.**

Ärztliche Versorgung in Pflegeheimen

1

a) Stimmen Sie der Aussage zu, dass die Versorgung von Heimbewohnern vom Hausarzt koordiniert werden sollte?

☐ nein ☐ ja

b) Sollte die Behandlung von Heimbewohnern durch andere Fachärzte in der Regel nur nach Überweisung durch Hausärzte erfolgen?

☐ nein ☐ ja

2

Wie schätzen Sie den Versorgungsbedarf durch folgende Arztgruppen ein?

	sehr gering 0	1	2	3	sehr hoch 4
Augenärzte	☐	☐	☐	☐	☐
Chirurgen/ Orthopäden	☐	☐	☐	☐	☐
Dermatologen	☐	☐	☐	☐	☐
Gynäkologen	☐	☐	☐	☐	☐
Hals-Nasen-Ohrenärzte	☐	☐	☐	☐	☐
Psychiater/ Neurologen	☐	☐	☐	☐	☐
Urologen	☐	☐	☐	☐	☐
Zahnärzte	☐	☐	☐	☐	☐

3

Inwieweit stimmen Sie den folgenden Aussagen zu?

	gar nicht 0	1	2	3	voll 4
Hausärzte sollten häufiger Besuche in Pflegeheimen durchführen	☐	☐	☐	☐	☐
Die Versorgung aller Bewohner eines Heimes nur durch eine Praxis führt (im Vergleich zu mehreren Praxen) zu einer höheren Qualität der Versorgung	☐	☐	☐	☐	☐
Speziell qualifizierte Medizinische Fachangestellte (MFA), die regelmäßig Heimbewohner besuchen, sind eine wichtige Unterstützung	☐	☐	☐	☐	☐
Bei der Versorgung von Heimbewohnern ist der Kassenärztliche Bereitschaftsdienst eine wichtige Unterstützung	☐	☐	☐	☐	☐
Für die medizinische Versorgung in Heimen können telemedizinische Konsile hilfreich sein	☐	☐	☐	☐	☐

4 **Wie schätzen Sie den Versorgungsbedarf durch folgende andere Berufsgruppen ein?**

	sehr gering 0	1	2	3	sehr hoch 4
Physiotherapeuten	☐	☐	☐	☐	☐
Logopäden	☐	☐	☐	☐	☐
Augenoptiker	☐	☐	☐	☐	☐
Hörgeräteakustiker	☐	☐	☐	☐	☐
Ernährungsberater	☐	☐	☐	☐	☐
Sozialarbeiter	☐	☐	☐	☐	☐

Versorgung im Krankenhaus und der Notaufnahme

5 **Inwieweit stimmen Sie den folgenden Aussagen zu?**

	gar nicht 0	1	2	3	voll 4
Von stationären Krankenhausaufenthalten profitieren Heimbewohner oftmals nicht	☐	☐	☐	☐	☐
Bei Heimbewohnern mit fortgeschrittener Demenz sollte die Indikation für einen Krankenhaustransport im Allgemeinen zurückhaltender gestellt werden	☐	☐	☐	☐	☐
Bei Stürzen von Heimbewohnern gibt es oft keine Alternative zu einem Transport ins Krankenhaus	☐	☐	☐	☐	☐
Pflegekräfte rufen zu häufig den Rettungsdienst ohne vorherige ärztliche Rücksprache	☐	☐	☐	☐	☐

6 **Insgesamt betrachtet, welchen Anteil stationärer Krankenhausaufenthalte und ambulanter Notaufnahmebesuche von Heimbewohnern schätzen Sie als nicht erforderlich ein?**

.... % der Krankenhausaufenthalte .... % der Notaufnahmebesuche

7 **Welche Bedeutung haben aus Ihrer Sicht die folgenden Maßnahmen, um Krankenhaustransporte von Heimbewohnern zu reduzieren?**

	keine 0	1	2	3	große 4
bessere Kommunikation zwischen Pflegekräften	☐	☐	☐	☐	☐
bessere Kommunikation zwischen Pflegekräften und Hausarzt	☐	☐	☐	☐	☐
bessere Versorgung/ Erreichbarkeit durch Hausarzt	☐	☐	☐	☐	☐
bessere Versorgung/ Erreichbarkeit durch Facharzt	☐	☐	☐	☐	☐
bessere Ausstattung des Heims (z.B. Katheter, Schnelltests, Medikamente)	☐	☐	☐	☐	☐
bessere Personalausstattung der Heime	☐	☐	☐	☐	☐
Qualifizierungsmaßnahmen für das Pflegepersonal	☐	☐	☐	☐	☐
aussagekräftige Patientenverfügung	☐	☐	☐	☐	☐
Sonstiges:	☐	☐	☐	☐	☐

Versorgung am Lebensende

8 **Bitte schätzen Sie die Situation in Deutschland ein:**

a) Wie hoch ist der Anteil an Heimbewohnern mit einer Patientenverfügung?

.................%

b) Wie häufig sind diese Verfügungen aussagekräftig in Bezug auf Krankenhaustransporte am Lebensende?

.................%

c) Wie häufig werden diese Verfügungen <u>nicht</u> dem Bewohnerwunsch entsprechend berücksichtigt?

.................%

9 **Inwieweit stimmen Sie den folgenden Aussagen zu?**

	gar nicht 0	1	2	3	voll 4
Heimbewohner werden am Lebensende zu häufig im Krankenhaus behandelt	☐	☐	☐	☐	☐
Hausärzte sind in der Regel gut für die Versorgung von Heimbewohnern am Lebensende ausgebildet	☐	☐	☐	☐	☐
Hausärzte sollten für die Versorgung am Lebensende auch außerhalb der Sprechzeiten erreichbar sein	☐	☐	☐	☐	☐
Pflegepersonal der Heime sollte besser für die Versorgung am Lebensende ausgebildet werden	☐	☐	☐	☐	☐
Heimbewohner müssten häufiger in die spezialisierte ambulante palliativmedizinische Versorgung (SAPV) eingeschrieben werden	☐	☐	☐	☐	☐

10 **Bitte schätzen Sie die Situation in Deutschland ein:**

a) Wie hoch ist der Anteil an Heimbewohnern, der im Krankenhaus und nicht im Heim verstirbt?

.... % der verstorbenen Heimbewohner

b) Wie hat sich der Anteil im Krankenhaus verstorbener Heimbewohner in den letzten 10 Jahren verändert?

☐ ist zurückgegangen ☐ blieb unverändert ☐ ist angestiegen

c) Wie ist die Versorgung von Heimbewohnern am Lebensende insgesamt?

☐ eher schlecht ☐ eher gut

→ **d) Falls Sie mit „eher schlecht“ geantwortet haben, was wäre nach Ihrer Einschätzung die wichtigste Maßnahme, mit der die Versorgung verbessert werden könnte?**

....

....

Versorgung durch Ärzte und andere Berufsgruppen in Ihrer Einrichtung

11 **Wie viele verschiedene Hausärzte versorgen mind. einen Bewohner in Ihrer Einrichtung?**

ca. .... Hausärzte

12 **Welchen Anteil der Bewohner, die im Krankenhaus stationär behandelt wurden, sieht der Hausarzt innerhalb der ersten Woche nach Entlassung?**

☐ 0-25% ☐ 26-50% ☐ 51-75% ☐ 76-100%

13 **Welche der folgenden Ärzte führen regelmäßig Hausbesuche in Ihrer Einrichtung durch (d.h. mind. ein Besuch pro Jahr bei mind. einem Bewohner)?**

	nein	ja	
Psychiater/ Neurologen	☐	☐	→ Dies sind ca. .... verschiedene Psychiater/ Neurologen.
Hals-Nasen-Ohrenärzte	☐	☐	→ Dies sind ca. .... verschiedene Hals-Nasen-Ohrenärzte.
Augenärzte	☐	☐	→ Dies sind ca. .... verschiedene Augenärzte.
Urologen	☐	☐	→ Dies sind ca. .... verschiedene Urologen.
Zahnärzte	☐	☐	→ Dies sind ca. .... verschiedene Zahnärzte.

14 **Welche der folgenden Berufsgruppen führen regelmäßig Hausbesuche in Ihrer Einrichtung durch (d.h. mind. ein Besuch pro Jahr bei mind. einem Bewohner)?**

	nein	ja	
Physiotherapeuten	☐	☐	→ Dies sind ca. .... verschiedene Physiotherapeuten.
Logopäden	☐	☐	→ Dies sind ca. .... verschiedene Logopäden.
Augenoptiker	☐	☐	→ Dies sind ca. .... verschiedene Augenoptiker.
Hörgeräteakustiker	☐	☐	→ Dies sind ca. .... verschiedene Hörgeräteakustiker.

Angaben zu Ihrer Einrichtung

15

a) **Trägerschaft:** ☐ Freigemeinnütziger Träger ☐ Privater Träger ☐ Kommunaler Träger

b) **Anzahl Pflegeplätze:** .... Betten

c) **In welchem Bundesland liegt die Einrichtung?** ....

d) **Wie groß ist der Ort, in dem die Einrichtung liegt?**

☐ bis 2.000 Einwohner ☐ bis 5.000 Einwohner ☐ bis 20.000 Einwohner

☐ bis 50.000 Einwohner ☐ bis 100.000 Einwohner ☐ mehr als 100.000 Einwohner

e) **Wie weit ist das nächste Krankenhaus mit Notaufnahme von Ihrer Einrichtung entfernt?** ca. .... km

Angaben zu Ihrer Person

16

a) **Alter:** .... Jahre

b) **Geschlecht:** ☐ männlich ☐ weiblich

c) **Ihre aktuelle Position:**

☐ Pflegedienstleitung ☐ Einrichtungsleitung ☐ Geschäftsführung

☐ sonstige, und zwar ....

d) **Wie lange sind Sie bereits in dieser Position tätig?** .... Jahre

Vielen Dank für Ihre Teilnahme!
